# Supplementary material for: Free energy and flexibility analysis of autoinhibited human BRAF
Source: bioRxiv. 2025 Aug 6:2025.08.04.668576. Preprint. [Version 1] doi: 10.1101/2025.08.04.668576 (PMC12340820; doi:10.1101/2025.08.04.668576)
Supplement: Supplement 1 [file NIHPP2025.08.04.668576v1-supplement-1.pdf]

bioRxiv preprint doi: https://doi.org/10.1101/2025.08.04.668576; this version posted August 6, 2025. The copyright holder for this preprint (which was not certified by peer review) is the author/funder. This article is a US Government work. It is not subject to copyright under 17 USC 105 and is also made available for use under a CC0 license.

Supplementary Information for

Free energy and flexibility analysis of autoinhibited human BRAF

Jeremy O. B. Tempkin[1,], Fikret Aydin[1,]*, Sebnem Essiz[2], Yue Yang[1], Timothy S. Carpenter[1], David E. Durrant[3], Deborah K. Morrison[3], Helgi I. Ingólfsson[1], Frederick H. Streitz[4], Dwight V. Nissely[5], Felice C. Lightstone[1], Xiaohua Zhang[1]*

[1]Physical and Life Sciences Directorate, Lawrence Livermore National Laboratory, Livermore, CA 94550, USA

[2]Molecular Biology and Genetics, Department of Engineering and Natural Sciences, Kadir Has University, Fatih 34083, Istanbul, Turkey

[3]Laboratory of Cell and Developmental Signaling, Center for Cancer Research (CCR), National Cancer Institute (NCI), Frederick, MD 21702, USA

[4]Computing Directorate, Lawrence Livermore National Laboratory, Livermore, CA 94550, USA

[5]NCI RAS Initiative, Cancer Research Technology Program, Frederick National Laboratory for Cancer Research, Leidos Biomedical Research, Inc., Frederick, MD 21702, USA

*Co-first authors and corresponding Authors: Jeremy O. B. Tempkin, tempkin1@llnl.gov, and Fikret Aydin, aydin1@llnl.gov.

bioRxiv preprint doi: https://doi.org/10.1101/2025.08.04.668576; this version posted August 6, 2025. The copyright holder for this preprint (which was not certified by peer review) is the author/funder. This article is a US Government work. It is not subject to copyright under 17 USC 105 and is also made available for use under a CC0 license.

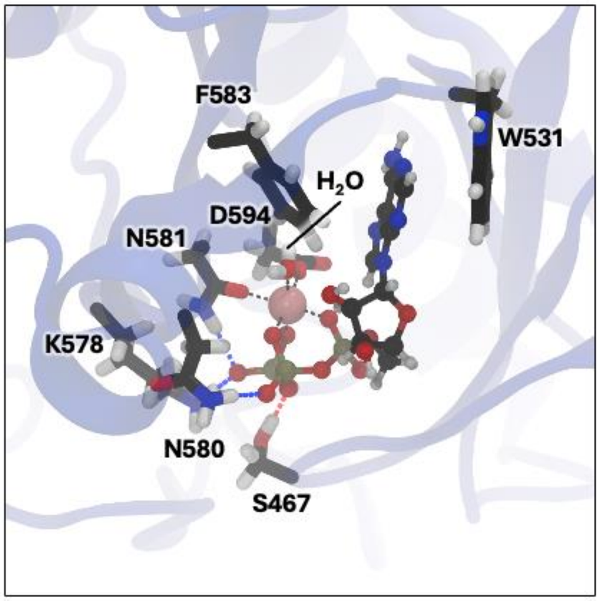

FIGURE S1. ATP binding site in KD. ATP and its coordinating Mg^{2+} ion were placed using MOE. ATP-Mg initial location and contacting amino acids were based on PDB 6U2G (chain B) followed by a short energy minimization to relax the ATP-Mg complex. The image shows the KD (blue), focused on the ATP binding pocket highlighting the ATP, Mg^{2+} (pink sphere) and contacting residues.

bioRxiv preprint doi: https://doi.org/10.1101/2025.08.04.668576; this version posted August 6, 2025. The copyright holder for this preprint (which was not certified by peer review) is the author/funder. This article is a US Government work. It is not subject to copyright under 17 USC 105 and is also made available for use under a CC0 license.

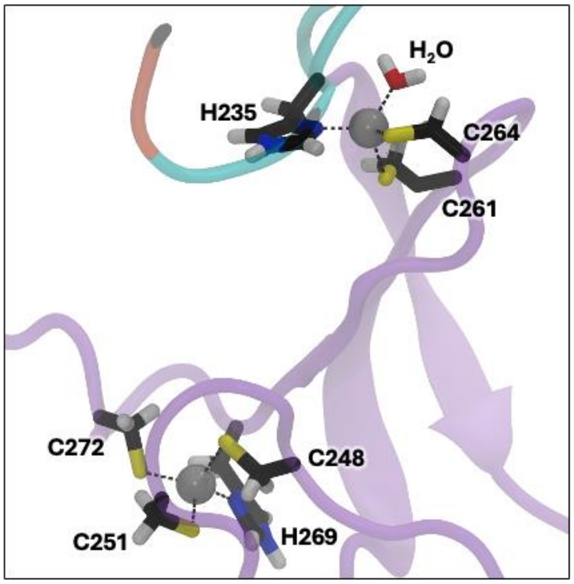

FIGURE S2. Zinc coordination sites in CRD. The reference structure PDB 7MFD does not resolve the fourth coordination of one of CRD's zinc (top). We completed the coordination of that zinc by adding a coordinating water molecule and placed it to ensure proper coordination of residue H235. The image shows the CRD's two zinc finger motifs highlighting the Zn^{2+} atoms and the contacting residues. The CRD is shown in purple, the C-terminal end of the RBD in orange, and the RBD-CRD linker in cyan. The two Zn^{2+} ions are shown in grey spheres.

bioRxiv preprint doi: https://doi.org/10.1101/2025.08.04.668576; this version posted August 6, 2025. The copyright holder for this preprint (which was not certified by peer review) is the author/funder. This article is a US Government work. It is not subject to copyright under 17 USC 105 and is also made available for use under a CC0 license.

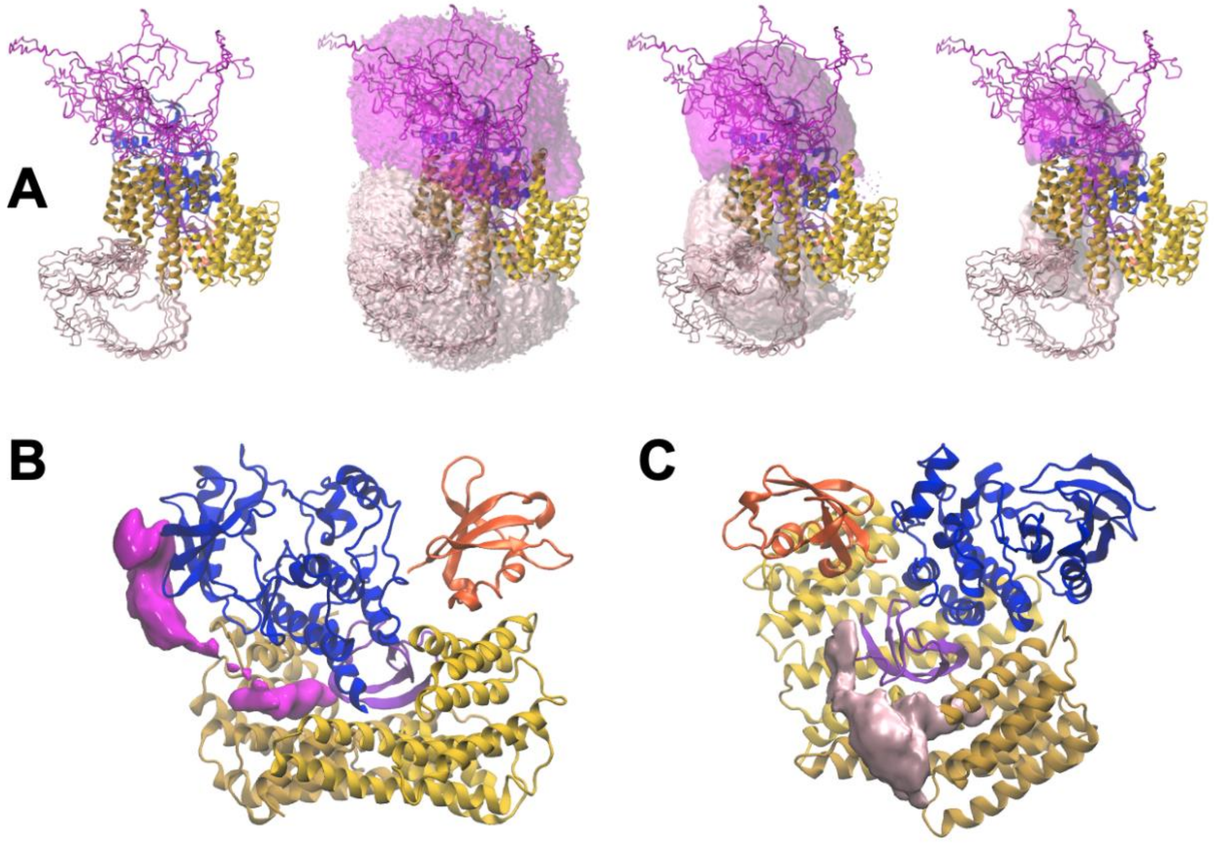

FIGURE S3. RAF loop placement and relaxation. Loops I (pink, residues 274 to 359) and II (magenta, residues 371 to 448) are disordered and are built into the model. The loops are built in with MOE and simulations are started with 27 different loop initial configurations, panel A. In the simulations the loops are very dynamic, sampling a larger confirmational space with the overall extent somewhat reduced compared to the initial placement, see example radius of gyration Fig. S4. A) The loops extended and common location are indicated with transparent loop density maps, showing low, medium, and high isovalues to illustrate the extended, commonly occupied, and frequently occupied areas for the loops. B) and (C) show zoomed in views of very high loop density areas for loop II and I, respectively, corresponding to where the loops are attached to the protein complex.

bioRxiv preprint doi: https://doi.org/10.1101/2025.08.04.668576; this version posted August 6, 2025. The copyright holder for this preprint (which was not certified by peer review) is the author/funder. This article is a US Government work. It is not subject to copyright under 17 USC 105 and is also made available for use under a CC0 license.

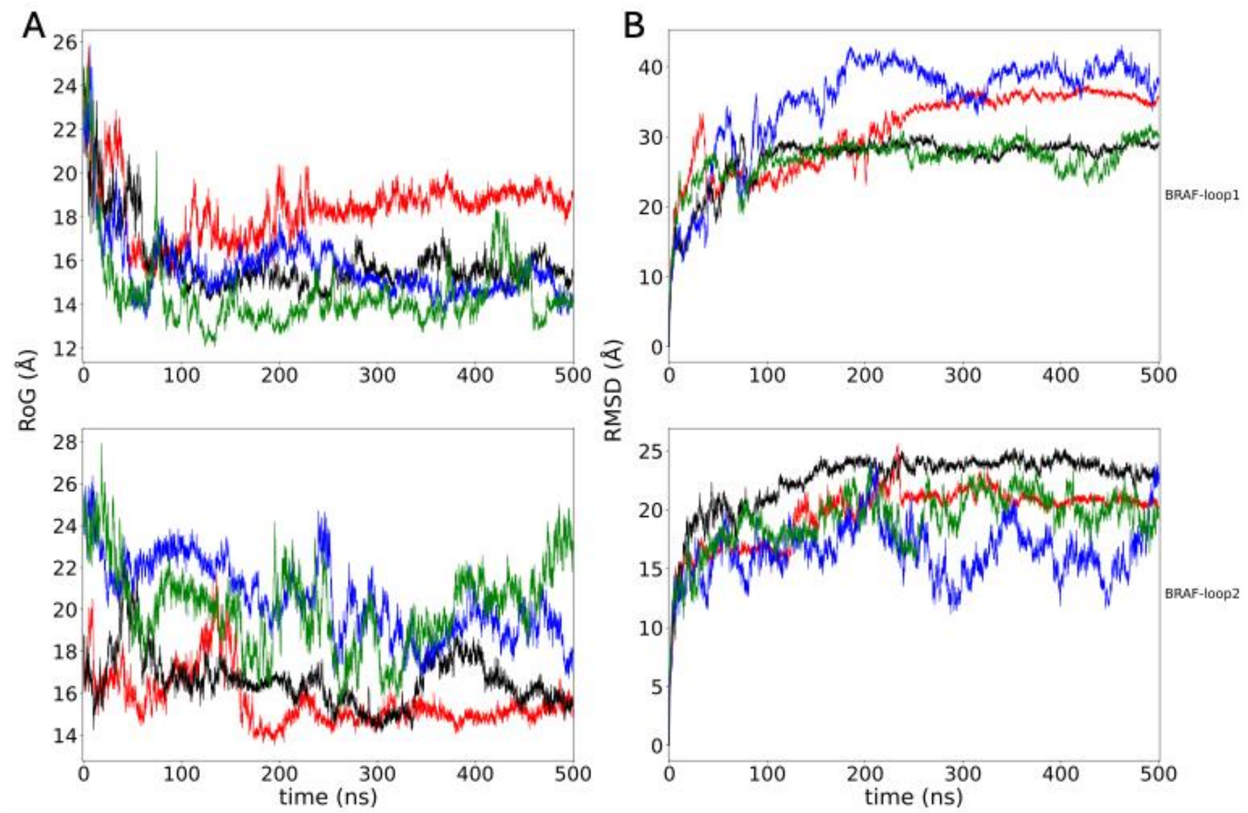

FIGURE S4. Extent and equilibration of the RAF loop configurations. Radius of gyration (RoG) (A) and RMSD (B) is shown for four random simulations of the 135 unbiased MD simulations. Top plots are for loop I (residues 282 to 359) and bottom plots for loop II (residues 371 to 448).

bioRxiv preprint doi: https://doi.org/10.1101/2025.08.04.668576; this version posted August 6, 2025. The copyright holder for this preprint (which was not certified by peer review) is the author/funder. This article is a US Government work. It is not subject to copyright under 17 USC 105 and is also made available for use under a CC0 license.

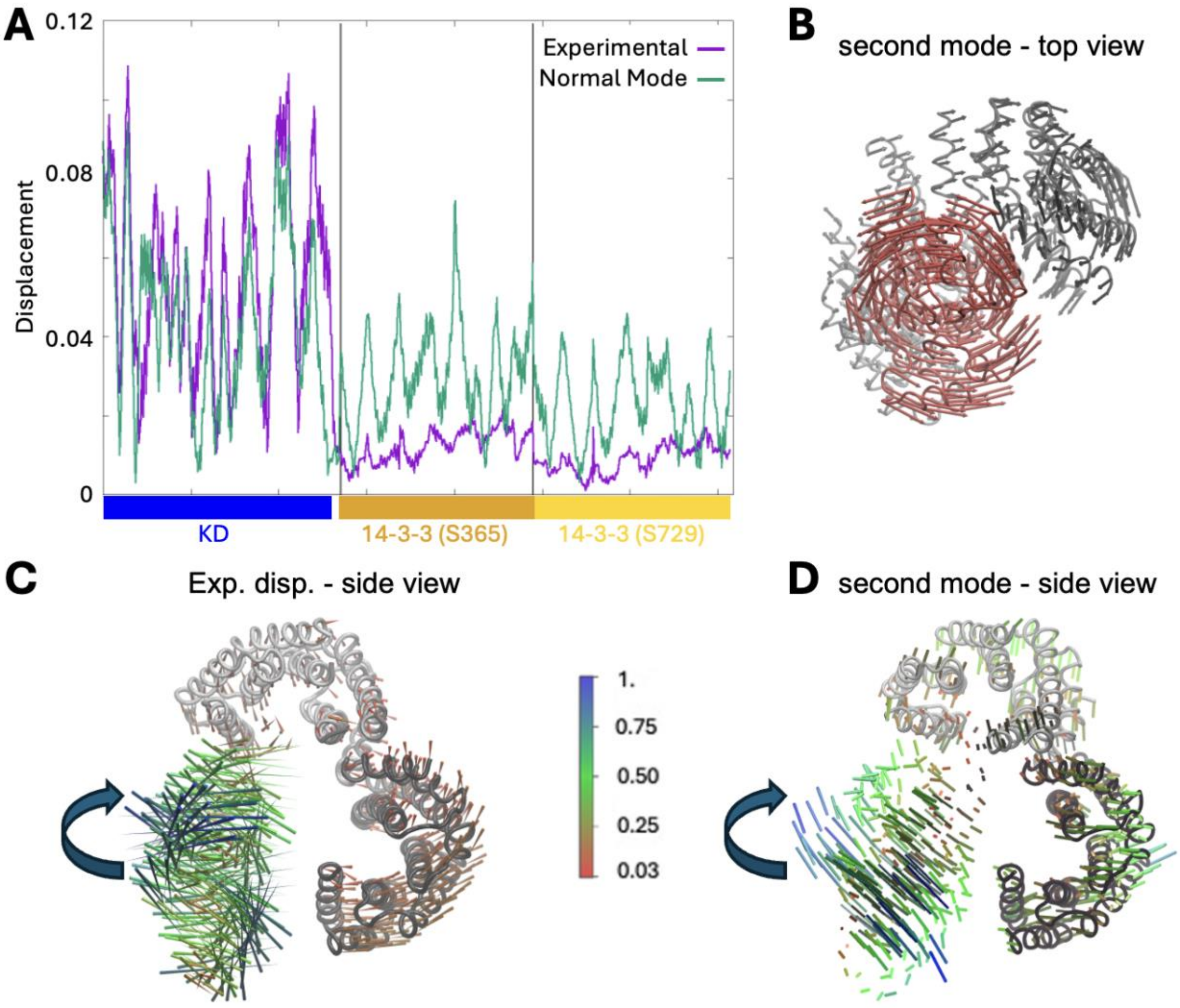

Figure S5. Details on the PCA analysis of the BRAF:14-3-3$_2$ complex. A) Comparison of the best overlapping mode (second PCA mode) with experimental conformational displacement. B) Displacement according to computed second mode, top view. C) Displacement of 14-3-3$_2$ and KD according to experimental conformational change PDB 7MFF to 7MFD. D) Displacement according to the computed second PCA mode. To assess whether the conformational flexibility around the autoinhibited state is related to the conformational changes required for the KD to transition between the monomeric and dimeric states, we compared these motions to the structural differences apparent between the dimerized BRAF (PDB: 7MFF) and monomeric BRAF (PDB:

bioRxiv preprint doi: https://doi.org/10.1101/2025.08.04.668576; this version posted August 6, 2025. The copyright holder for this preprint (which was not certified by peer review) is the author/funder. This article is a US Government work. It is not subject to copyright under 17 USC 105 and is also made available for use under a CC0 license.

7MFD) cryo-EM structures. We first superimposed the KD and 14-3-3 dimer from the monomeric BRAF structure 7MFD onto the KD and 14-3-3_2 from the dimeric structure 7MFF. The RBD and CRD segments are discarded from 7MFD structure as well as from the PCA modes as these domains are not resolved in the dimer structure. Then we calculated the unit normalized displacement vector between 7MFD and 7MFF and compared the linear displacement required to transform the KD and 14-3-3_2 complex. We show that the similarity of the vector is checked against the largest amplitude PCA modes. The second mode has a rotation type of movement for kinase. This second mode displayed highest similarity (dot product of the two vectors is -0.44) to the conformational change in between the cryo-EM structures. In panel A we show the experimental displacement vector together with the second PCA mode; 14-3-3_2 domain is more mobile in the PCA mode, while the KD region displacements seem similar. In panel B the same PCA vector is projected onto the structure with arrows from top view of the rotation axis of the kinase. The rotational motion of kinase around the same axis is visible in experimental displacement in between 7MFD and 7MFF (panel C) and the second mode obtained from the PCA analysis of the MD trajectory (panel D).

bioRxiv preprint doi: https://doi.org/10.1101/2025.08.04.668576; this version posted August 6, 2025. The copyright holder for this preprint (which was not certified by peer review) is the author/funder. This article is a US Government work. It is not subject to copyright under 17 USC 105 and is also made available for use under a CC0 license.

Table S1. Details on the domains/residues in the BRAF:14-3-3$_2$ model. Column 1 lists the names of each domain/region. Columns 2 and 3 list the starting and ending residue numbers for each region respectively. Column 4 describes whether the position of those residues were determined from the cryo-EM structure (PDB: 7MFD) or added to the structure by the modeling approaches described in the main text.

Domain/region	Start residue	End residue	Cryo-EM or modeled?
RBD	156	201	Cryo-EM
	202	*203*	*Modeled*
	204	227	Cryo-EM
RBD-CRD linker	*228*	*234*	*Modeled*
CRD	235	273	Cryo-EM
CRD-zinc loop	*274*	*281*	*Modeled*
Loop I	*282*	*359*	*Modeled*
pS365 region	360	370	Cryo-EM
Loop II	*371*	*448*	*Modeled*
	449	456	Cryo-EM
KD	457	723	Cryo-EM
PS729 region	724	738	Cryo-EM
14-3-3 zeta (S365)	2	69	Cryo-EM
	70	*72*	*Modeled*
	73	203	Cryo-EM
	204	*209*	*Modeled*
	210	230	Cryo-EM
14-3-3 zeta (S729)	2	69	Cryo-EM

bioRxiv preprint doi: https://doi.org/10.1101/2025.08.04.668576; this version posted August 6, 2025. The copyright holder for this preprint (which was not certified by peer review) is the author/funder. This article is a US Government work. It is not subject to copyright under 17 USC 105 and is also made available for use under a CC0 license.

	70	*71*	*Modeled*
	72	133	Cryo-EM
	134	*136*	*Modeled*
	137	230	Cryo-EM
